# CAR T Cell Therapy of Non-hematopoietic Malignancies: Detours on the Road to Clinical Success

**DOI:** 10.3389/fimmu.2018.02740

**Published:** 2018-12-03

**Authors:** Kristen B. Long, Regina M. Young, Alina C. Boesteanu, Megan M. Davis, J. Joseph Melenhorst, Simon F. Lacey, David A. DeGaramo, Bruce L. Levine, Joseph A. Fraietta

**Affiliations:** ^1^Department of Biology, Mansfield University, Mansfield, PA, United States; ^2^Center for Cellular Immunotherapies, Abramson Cancer Center, University of Pennsylvania, Philadelphia, PA, United States; ^3^Parker Institute for Cancer Immunotherapy, University of Pennsylvania, Philadelphia, PA, United States; ^4^Department of Pathology and Laboratory Medicine, Perelman School of Medicine, University of Pennsylvania, Philadelphia, PA, United States

**Keywords:** CAR T cell, immunotherapy, cancer, solid tumor, microenvironment, adoptive cell therapy, non-hematopoietic malignancy

## Abstract

Chimeric antigen receptor (CAR)-engineered T cells represent a breakthrough in personalized medicine. In this strategy, a patient's own T lymphocytes are genetically reprogrammed to encode a synthetic receptor that binds a tumor antigen, allowing T cells to recognize and kill antigen-expressing cancer cells. As a result of complete and durable responses in individuals who are refractory to standard of care therapy, CAR T cells directed against the CD19 protein have been granted United States Food and Drug Administration (FDA) approval as a therapy for treatment of pediatric and young adult acute lymphoblastic leukemia and diffuse large B cell lymphoma. Human trials of CAR T cells targeting CD19 or B cell maturation antigen in multiple myeloma have also reported early successes. However, a clear and consistently reproducible demonstration of the clinical efficacy of CAR T cells in the setting of solid tumors has not been reported to date. Here, we review the history and status of CAR T cell therapy for solid tumors, potential T cell-intrinsic determinants of response and resistance as well as extrinsic obstacles to the success of this approach for much more prevalent non-hematopoietic malignancies. In addition, we summarize recent strategies and innovations that aim to augment the potency of CAR T cells in the face of multiple immunosuppressive barriers operative within the solid tumor microenvironment. Advances in the field of CAR T cell biology over the coming years in the areas of safety, reliability and efficacy against non-hematopoietic cancers will ultimately determine how transformative adoptive T cell therapy will be in the broader battle against cancer.

## Introduction

The use of genetically engineered T cells as a form of cancer therapy heralds a new era of synthetic biology and medicine. Within the past few years, clinical trials using chimeric antigen receptor (CAR) T cells to recognize and eliminate hematopoietic malignancies have demonstrated high rates of response as well as durability of remission that are unprecedented in ALL (1–3), chronic lymphocytic leukemia (CLL) (4, 5), and refractory B cell lymphomas (6, 7). This culminated in the recent United States Food and Drug Administration approvals of CD19-directed CAR T cells for relapsed/refractory pediatric and young adult ALL and diffuse large B cell lymphoma (DLBCL). While CAR T cell therapy is poised to revolutionize the treatment of leukemias and lymphomas, the field awaits a clear demonstration of efficacy against non-hematopoietic malignancies. The key challenges for these immunotherapies are how to: (I) safely enhance the potency and sustain the function of CAR T cells *in vivo* and (II) develop mechanism-based strategies to increase the resistance of CAR T cells to intrinsic and extrinsic dysfunction. Advances in basic and translational research aimed at improving the safety, consistency and effectiveness of CAR T cells against tumors of non-hematopoietic origin will ultimately determine whether this approach can find wider applications in cancer as well as other diseases.

Adoptive cellular immunotherapy involves expanding T cells from a patient or donor *in vitro*, followed by reinfusion of tumor-specific lymphocytes as cancer therapy. Transfer of expanded tumor infiltrating lymphocytes (TILs) from a subset of individuals with metastatic melanoma has shown potent anti-tumor effects (8, 9). It is likely that TILs target neoantigens within the broad landscape of mutant peptides encoded by *de novo* somatic mutations (10–14). In rare instances, adoptive transfer of autologous T cells targeting antigens encoded by somatically mutated genes has also resulted in clinically meaningful regressions of colon, metastatic bile duct, cervical and breast cancers (15–19). However, this strategy has little effect on other common epithelial malignancies that have lower mutation rates.

Transfer of genetically-redirected T cells bypasses many of the mechanisms involved in immunological tolerance by the creation of antigen-specific lymphocytes independently of intrinsic tumor immunogenicity that is driven at least in part by a high mutational burden. T cells can be directed to novel tumor antigens by introducing genes encoding new antigen receptors, including natural T cell receptors (TCRs) and CARs. CARs are synthetic molecules that combine the effector functions of T cells with the ability of antibodies to detect pre-defined antigens with a high degree of specificity in a non-major histocompatibility complex (MHC) restricted manner (20). These receptors can therefore recognize intact proteins and do not rely on endogenous antigen processing and presentation. CARs are typically comprised of an extracellular domain for tumor recognition and an intracellular signaling domain that mediates T cell activation [reviewed in 21–24)]. The antigen-binding function of a CAR is usually conferred by a single chain variable fragment (scFv) containing the variable heavy (V_H_) and variable light (V_L_) chains of an antibody fused to peptide linker (20, 25, 26). This extracellular portion of the receptor is fused to a transmembrane domain followed by intracellular signaling modules. First-generation chimeric receptors bearing CD3ζ alone were not sufficient to elicit proliferation or cytokine production in peripheral T cells (27), which likely explains their failure to consistently expand and persist in some of the earliest clinical trials of CAR T cells (28, 29). However, the incorporation of co-stimulatory endodomains into CARs can recapitulate natural co-stimulation (30–32). We and others have demonstrated remarkable rates of complete and durable remission in patients with CLL (4, 5, 33), ALL (1–3), and Non-Hodgkin lymphomas (6, 7, 34) treated with second-generation CD19-directed CARs incorporating 4-1BB or CD28 co-stimulation. Early clinical trials of CAR T cells for the treatment of multiple myeloma have also demonstrated promising results (35–37). Thus, in the setting of hematopoietic malignancies, CAR T cells are emerging as a powerful therapy with the curative potential of allogeneic stem cell transplantation, but without the acute and chronic toxicity of graft-vs.-host disease and conditioning regimens. In contrast, CAR modified T cells are less effective than immune checkpoint blockade and in some cases TIL-based immunotherapy in treating patients with solid tumors to date. In this review, we will discuss the history and current status of CAR T cell therapy for non-hematopoietic malignancies, outline intrinsic mechanisms of T cell potency, describe extrinsic barriers operative in the setting of treating solid tumors, and suggest strategies to enhance the effectiveness of this approach for a variety of these incurable cancers.

## History and current status of Car T cell therapy for non-hematopoietic cancers

### Initial clinical trials of Car T cell therapy in solid tumors

In early clinical trials of first-generation CAR T cells for solid tumors, safety and therapeutic efficacy were difficult to determine because of the aforementioned poor *in vivo* expansion and persistence of the transferred lymphocytes. These studies included patients with advanced epithelial ovarian cancer or metastatic renal cell carcinoma and targeted the folate receptor or carbonic anhydrase IX (CAIX), respectively (28, 29). A clinical trial of L1-cell adhesion molecule-specific (CD171) CAR T cells for the treatment of metastatic neuroblastoma demonstrated similar results of short-persisting (1–7 days) CAR T cells in individuals with bulky disease, but significantly longer persistence (42 days) in a single patient with limited tumor burden (38). Later trials of first-generation GD2-targeted CAR T cells administered to children with advanced neuroblastoma were more encouraging, with 3 of 11 patients experiencing complete remission, no substantial toxicity observed and sustained therapeutic benefit reported for several subjects (39, 40). Although the results of these trials were encouraging and provided the impetus to incorporate co-stimulatory signaling motifs in addition to CD3ζ, a third-generation CAR specific to the tumor antigen Her2 and integrating CD28, 4-1BB, and CD3ζ signaling moieties resulted in death of a patient with metastatic colon cancer (41). In this case, toxicity was caused by on-target, off-tumor reactivity of the CAR T cells with Her2 on normal lung and/or cardiac tissue (41). This serious adverse event was likely attributed to the infusion of substantially higher numbers of CAR T cells following lymphodepleting chemotherapy compared to most other trials. A second-generation Her2 CAR was also tested in patients with sarcoma without evidence of toxicity (42). Although there were some indications of anti-tumor activity in this trial, T cell persistence was limited, similar to earlier clinical studies.

### Recent clinical studies of Car T cell therapy in non-hematopoietic malignancies

Less dramatic clinical responses have also been observed in recently conducted clinical trials designed for the treatment of solid tumors with CAR T lymphocytes. Although evaluable data are not yet available from many of these studies, there is enough proof-of-concept from successful human studies of CAR T cells in leukemia and lymphoma to establish a concrete platform to treat these other indications. A complete response to CAR T cell therapy of recurrent multifocal glioblastoma was achieved using autologous T cells genetically-redirected to the tumor-associated antigen interleukin-13 receptor alpha 2 (IL13Rα2) (43). Interestingly, multiple intracavitary and intraventricular administrations of IL13Rα2 CAR T cells induced increases in the frequencies and absolute numbers of endogenous immune cells (i.e., CD3^+^ T cells, CD14^+^ CD11b^+^ HLA-DR^+^ mature myeloid populations, CD19^+^ B cells, and few CD11b^+^ CD15^+^granulocytes) in association with the elaboration of inflammatory cytokines. This case underscores the possible role of the endogenous immune system in potentiating the anti-tumor activity of engineered CAR T cells and the potential of this approach to safety and dramatically increase quality of life in patients with malignant brain tumors (43).

**Figure 1:**
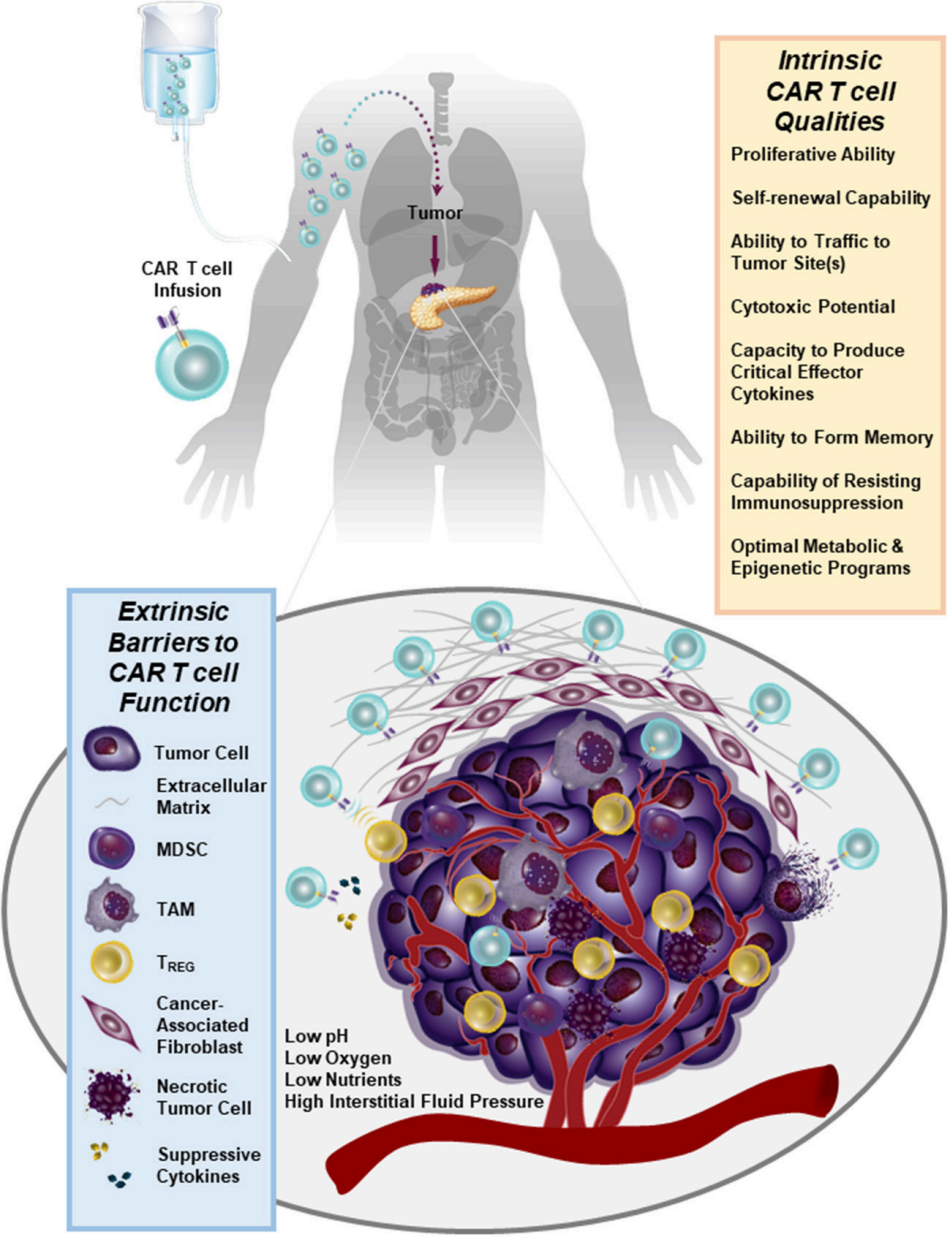
Numerous immunosuppressive barriers present in the solid tumor microenvironment that can hamper the efficacy of CAR T cell therapy are schematically depicted. Several intrinsic qualities of CAR T cells that may impact the anti-tumor potency of these lymphocytes are also listed.

We have recently generated CARs directed against the epidermal growth factor receptor variant III (EGFRvIII) and used them to gene engineer glioblastoma multiforme (GBM)-specific T cells. We found that we can redirect GBM patient T cells to target glioma tumors via lentiviral transduction with a CAR recognizing EGFRvIII *in vitro*, as well as *in vivo* in murine models (44) and in 10 patients (45) without the systemic toxicity associated with current standard-of-care treatments. In our first-in-human trial of EGFRvIII CAR T cells, we were able to confirm that a single intravenous infusion of these modified lymphocytes resulted in T cell engraftment in the peripheral blood, trafficking to the brain and antigen-directed activity (45). However, we observed that the inhibitory tumor microenvironment ultimately hampers clinical efficacy: following CAR T cell administration, several immunosuppressive factors were upregulated in the tumor environment including programmed death-ligand 1 (PD-L1), tryptophan 2,3-dioxygenase, indoleamine 2,3-dioxygenase, and IL-10. The lack of CAR T cell anti-tumor activity was accompanied by the presence of immunosuppressive regulatory T cells (T_REGS_) based on their expression of CD4, CD25, and FoxP3. Furthermore, the heterogeneity of EGFRvIII expression was a clear barrier to ongoing clinical responses in this study (45). Thus, adoptive cell therapies for non-hematopoietic malignancies will need to address how to increase both the potency and persistence of CAR T cells in the face of antigen heterogeneity and a strongly suppressive tumor microenvironment (Figure 1). This clinical report (45) presents several known obstacles to CAR T cell therapy for solid tumors which are described below in detail.

## Tuning Car T cell specificity and intrinsic fitness for immunotherapy of solid tumors

### Tumor antigen expression and heterogeneity

**Figure 2:**
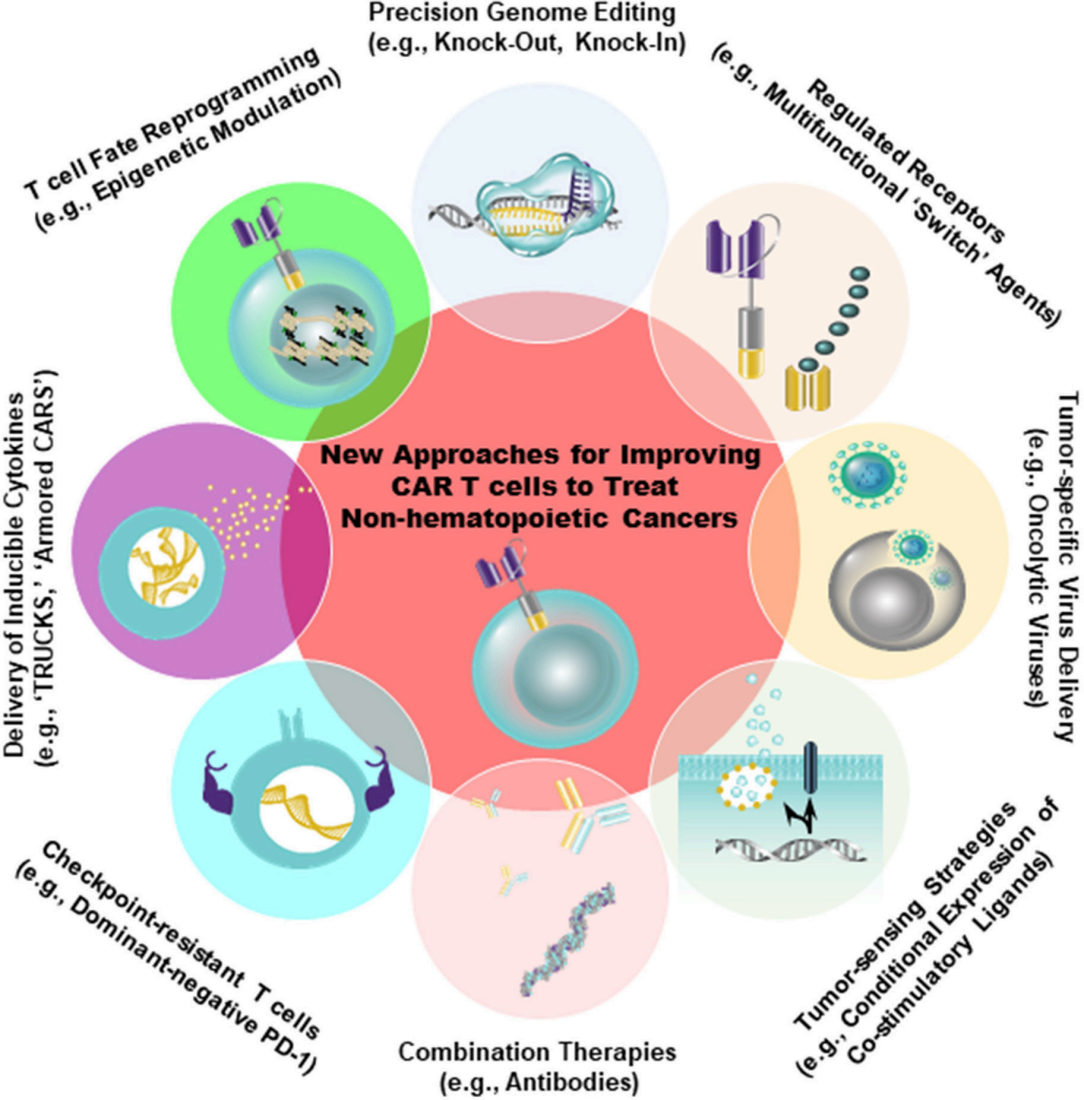
Strategies to improve the safety (e.g., tumor-sensing strategies) as well as to augment the anti-tumor efficacy of CAR T cells are shown. Genetic engineering can be accomplished using viral (e.g., lentiviruses, retroviruses) and non-viral (e.g., CRISPR/Cas9) approaches to endow CAR T cells with gain-of-function or loss-of-function alterations. The overall aim of these approaches is to improve intrinsic T cell fitness and allow these cells to elicit optimal effector activity in the setting of several extrinsic barriers operative within solid tumors, as shown in Figure 1.

Despite the fact that antigens such as CD19 and B-cell maturation antigen (BCMA) have been successfully targeted by CARs in the setting of hematopoietic cancer, there is an unmet need to identify similarly ideal antigens expressed by solid tumors. A major barrier to the development of CARs for solid tumor indications is, indeed, the identification of tumor antigens that can be targeted safely and effectively [reviewed in 46). In an optimal setting, CAR T cells should be directed against a tumor-restricted antigen to avoid on-target, off-tumor reactivity with healthy tissues. The proposed target antigen should be differentially expressed on tumor cells relative to essential normal tissues. In addition, the chimeric receptor must be highly specific for an antigen that is broadly expressed on the majority of cancer cells (46, 47). A variety of tumor-specific and tumor-associated antigens that can be targeted using CAR T cell therapy in non-hematopoietic malignancies have been identified (e.g., EGFR/EGFRvIII, IL13Rα2, Her2, CD171, mesothelin (MSLN), folate receptor alpha, GD2, carcinoembryonic antigen (CEA), chondroitin sulfate proteoglycan 4, c-Met, etc.). Antigens that display high constitutive expression that is tumor-restricted (e.g., chondroitin sulfate proteoglycan 4) may permit the application of CAR T cell therapy to higher proportions of patients and reduce the likelihood of tumor escape (48). However, because most tumor-associated antigens are heterogeneously expressed in tumor tissue, the efficacy of CAR T cells is often limited. Thus, combination therapies incorporating CARs that target multiple antigens will likely be required. There is progress in more safely and specifically targeting non-hematopoietic tumors with CAR T cells, either through creating CAR T cells specific for RNA splice variants or tumor-specific glycans (49, 50), or by generating CAR T cells that are conditionally specific for solid tumors. The latter is achieved by employing sensing and switching strategies in the tumor microenvironment (51–54). In addition to selectively replicating in and killing tumor cells directly, oncolytic viruses armed with payloads (e.g., bispecific T cell engagers, cytokines) may further synergize with CAR T cells to overcome tumor heterogeneity, while simultaneously bolstering anti-tumor activity (55, 56) (Figure 2).

### Car T cell trafficking to solid tumors

Following infusion of CAR T cells targeting an appropriate antigen into patients, these lymphocytes are faced with the immediate obstacle of having to successfully localize to the tumor bed. This process is critically dependent on chemokine receptors expressed by the transferred cells and the chemokine gradient produced by the tumor. This presents a challenge because T cells often do not express the cognate receptors for the chemokines produced by tumors. In addition to this chemokine/chemokine receptor mispairing, tumors produce very small amounts of the chemokines needed for successful trafficking of T cells to the lesion. For example, melanoma cells do not produce sufficient amounts of CXCR3 ligands and this results in inefficient localization of CXCR3 receptor-bearing effector CD8^+^ T cells to metastatic sites 57. We and others have co-expressed better matched chemokine receptors with CARs which resulted in improved trafficking of CAR T cells and enhanced tumor elimination (58, 59).

### Characteristics of intrinsic Car T cell potency

Systematic evaluations of patients with hematologic malignancies responding or not responding to CAR T cell therapy has yielded insights into key determinants of T cell potency that may inform treatment of solid tumors. In CLL, CAR T cells that were particularly effective exhibited robust proliferative capacity as well as long-term persistence *in vivo*. Transcriptomic profiling of patient-derived cell products revealed that CAR T cells from complete-responding patients were enriched in memory related genes, including IL-6/STAT3 signatures, whereas products from non-responding patients upregulated programs involved in effector T cell differentiation, glycolysis, exhaustion, and apoptosis (33). Unexpectedly, there was no association with typical patient-(e.g., age, sex, prior therapy) or disease-related (prior therapies, genetic and other risk profile, tumor burden, etc.) factors with likelihood of response. This makes the important point that cell-intrinsic properties are major determinants of success and failure in CAR T cell therapy (Figure 1).

### Generation of quality Car T cells

The optimal “seed” population of T cells needed for the generation of CAR T cells that can sustain durable responses against cancer is still a matter of debate. One school of thought is that effector CD8^+^ T cells producing high amounts of interferon-gamma are most effective at eliminating tumors, while other investigators believe that naïve or early memory CD8^+^ T cells which differentiate and expand at the tumor site are superior for eliciting long-lasting anti-tumor immunity (60–62). If one assumes a linear model of CD8^+^ T cell differentiation, naïve T lymphocytes (T_N_) are programmed into the earliest identifiable memory T cell stage, stem cell memory (T_SCM_). This population is thought to give rise to the successive stages of differentiation: central memory (T_CM_), effector memory (T_EM_), terminally differentiated effector memory RA (T_EMRA_), and effector (T_EFF_) cells (63). Many studies have supported the idea that early memory CD8^+^ T cells generate the most potent CAR T cells against both liquid and solid tumors. For example, CAR-engineered T_SCM_ cells directed to mesothelin were significantly more effective at regressing established solid tumors compared to T_EM_ and T_EFF_ cells (63). Retrospective profiling of *ex vivo* CD4^+^ and CD8^+^ T cells from CLL patients treated with anti-CD19 CAR T cells revealed that responding and non-responding patients did not differ in their frequencies of T_N_, T_CM_, T_EM_, or T_EFF_ cells at the time of T cell collection. However, responding patients did exhibit a modest increase in T_SCM_ cells compared to non-responders (33). More significantly, unbiased biomarker analysis revealed that the frequency of apheresed CD27^+^ CD45RO^−^ CD8^+^ T cells from patients responding to CAR T cell therapy was significantly higher compared to non-responder T cells. Notably, this subpopulation of CD8^+^ T cells possessed functional characteristics of early memory as well as effector T cells (33).

Based on growing pre-clinical and clinical evidence of less-differentiated cells mediating superior anti-tumor efficacy, there is interest in developing ways to conduct large-scale T cell expansion, while simultaneously preserving the functional features of early-memory T cells. Human T cells undergo a series of profound changes with successive rounds of division *in vitro* and *in vivo*. Among these changes are the loss of certain co-stimulatory receptors (e.g., CD28, CD27) and the erosion of telomeres. Depending on the molecular design, co-stimulatory endodomains from these receptors may or may not be incorporated into the CAR. Therefore, culture systems that can prevent telomere loss or potentiate the maintenance of endogenous co-stimulatory receptor expression could restore proliferative potential to conventional effector T cells and presumably increase the functional lifespan of these cells following re-infusion into patients (64, 65). We have recently described a culture system for the production of CAR T cells in 3–5 days, relative to a traditional 9-day process (66). This process allowed us to generate CD19-directed CAR T cells that were less differentiated and, at limited cell doses, significantly more potent against leukemia in an *in vivo* animal model (66). Alternative approaches for reducing CAR T cell differentiation during *in vitro* expansion include inhibition of signaling mediators downstream of the IL-2 pathway such as subunits of Glycogen synthase kinase 3β (60), Protein kinase B (AKT) (67), and Phosphoinositide 3-kinase (68). In addition, replacement of IL-2 with other cytokines such as IL-7 and IL-15 that signal through the γ-common chain receptor (69), but regulate survival and homeostatic T cell proliferation independently of TCR stimulation (70–72) may enhance the *in vivo* expansion and persistence of CAR T cells (73, 74). Genetic reprogramming of induced pluripotent stem cells derived from somatic cells could also be used to generate more naïve-like CAR T lymphocytes for adoptive transfer (75). Finally, in a “bedside-to-bench” study, we demonstrated that unintentional disruption of the gene encoding the methylcytosine dioxygenase TET2 resulted in the massive clonal expansion of CAR T cells that were all derived from a single cell. Furthermore, *TET2*-disrupted lymphocytes exhibited a predominantly T_CM_ phenotype at the peak of the anti-tumor response (76). These findings, along with other recent reports (77–81), underscore the power of epigenetic modulation in effectively re-programming T lymphocyte fate for the generation of CAR T cells with optimal anti-tumor potency (Figure 2).

## Surmounting tumor-mediated barriers to Car T cell therapy of non-hematopoietic cancers

A major issue to be addressed for improving the efficacy of CAR T cells against non-hematopoietic malignancies is determining how to effectively enhance the persistence and function of these lymphocytes in toxic tumor microenvironments. CAR T cells are vulnerable to both immunological and metabolic checkpoints as well as other suppressive factors present in the tumor bed. In pre-clinical mouse models, both CAR and TCR transgenic T cells cease to function or die shortly after entering the tumor microenvironment (82, 83). Although repeated infusions of freshly engineered T cells may help to improve engraftment, this approach is not always clinically feasible. Tumor-imposed extrinsic barriers as well as strategies to overcome several of these hurdles for the generation of efficacious CAR T cells to treat solid cancers are described below.

### Overcoming physical barriers in solid tumors

Unlike liquid tumors which do not typically possess physical barriers that would prevent their interactions with CAR T cells, many solid tumors have a formidable barricade that renders these masses inaccessible to invasion by immune cells. This landscape includes stromal cells, immune cells, cancer cells and extracellular matrix (ECM) components (i.e., proteins and glycans). Collagens, fibronectin, laminin, hyaluronan, and proteoglycans heavily contribute to the proliferation of fibrous or connective tissue (desmoplasia). The fibrotic tumor stroma of many solid malignancies, including pancreatic, breast and ovarian cancer is thought to impede effective drug delivery (84–86) and may also prevent infiltration by CAR T cells (Figure 1). Accordingly, diffusion of the CAR T cells into tumor tissue was shown to be blocked by the ECM are therefore often trapped (87) and unable to deeply penetrate tumor tissue (88). Desmoplasia combined with high interstitial fluid pressure and rapid tumor cell proliferation also contributes to the collapse of vasculature, which may further impede CAR T cell infiltration from vessels into tumor tissue (89). Tumor vessels may also not possess the receptors necessary for T cell homing and extravasation, including E- and P-selectins, VCAM-1, and ICAM-1 (87). Furthermore, following *in vitro* culture, CAR T cells often lack normal expression of the enzyme heparanase which degrades matrix proteoglycans and potentiates extravasation (90).

Administration of collagenases or hyaluronidase into solid tumors has been shown to enhance ECM breakdown, rendering the tumor more penetrable and thus susceptible to drug and cell-based therapies. Collagenase or hyaluronidase treatment has aided in increased antibody diffusion and chemotherapy uptake in pre-clinical *in vivo* and *in vitro* models of disease (91–94). Alternatively, reprogramming of myeloid cells, which naturally traffic and infiltrate into solid tumors, can effect anti-fibrotic activity and ECM breakdown (95). Depletion of ECM-producing cells (e.g., cancer-associated fibroblasts) can also render solid tumors more susceptible to therapy (96). In this regard, targeting stromal fibroblasts with anti-fibroblast activation protein (FAP) CAR T cells significantly stalls the growth of multiple types of solid tumors (97). In addition, administration of CAR T cells engineered to overexpress heparanase leads to partial ECM degradation, enhanced T cell infiltration and anti-tumor activity (90). Although these strategies seem promising, the potential negative impact of tumor ECM depletion should not be overlooked. In some studies, ECM reduction can paradoxically accelerate disease progression (98, 99). To avoid this potential negative outcome, direct intracavitary or intratumoral injection relative to intravenous infusion of CAR T cells may circumvent many of the physical barriers described above. In this vein, Klampatsa et al. used intracavitary methods to eliminate mesothelioma cell lines with some success (100), and Adusumilli and colleagues demonstrated that intrapleural administration of CAR T cells was significantly more successful at eliciting anti-tumor activity than the intravenous route (101).

### Targeting the tumor vasculature and immune stimulatory Car T cell modifications

In addition to tumor antigens, CARs can be targeted to the tumor vasculature in an effort to restrict blood flow and nutrient supplies to the tumor, which impedes malignant growth and simultaneously increases T cell localization (102). A strategy based on regional infusion of IL-12 secreting CAR T cells directed against VEGFR-2 which is expressed on angiogenic endothelial cells resulted in enhanced accumulation of these lymphocytes and tumor regression in multiple pre-clinical models (103). “Armored CARs” or “TRUCKs” (T cells Redirected for Universal Cytokine Killing) delivering other cytokines such as IL-15 (104, 105) or IL-18 (106) to the tumor microenvironment have also demonstrated superior anti-tumor activity compared to conventional CAR T cells (Figure 2). Furthermore, echistatin CARs targeting the angiogenic integrin αvβ3, which is commonly expressed on vascular endothelium of solid tumors (107), increased nanoparticle deposition in tumors (108). These findings indicate that the use of vasculature-targeted CAR T cells may be a potential “lead-in” strategy to enhance delivery of drugs or other adoptively transferred immune cells.

### Overcoming cell-mediated immunosuppression in the solid tumor microenvironment

Along with physical barriers, the tumor microenvironment is composed of multiple cellular components and molecular factors that can abrogate the elicitation of effective endogenous anti-tumor immune responses. This immunosuppressive milieu can also severely inhibit the effector functions of adoptively transferred CAR T cells. However, CAR T cell hypofunction is tightly dependent on the tumor microenvironment and in some instances removal of engineered T cells from the tumor restores their functional activity (109). This report as well as other studies (110–112) suggest that favorably altering the toxic tumor microenvironment by directly targeting immunosuppressive cells or engineering T cells to resist tumor-specific inhibitory mechanisms may provide new opportunities to improve CAR T cell function.

Tumor associated macrophages (TAMs) are an immunosuppressive cell type commonly found in solid tumors, and these cells aid in tumor cell survival and growth. While the phenotype of macrophages is pliable and these cells can be programmed to be either tumor-promoting or tumor-suppressive, macrophage function is ultimately dictated by signals from the surrounding tissue-specific niche (113). The tumor microenvironment often pushes macrophages toward a tumor-promoting phenotype (114), and this aids in angiogenesis, growth, immune evasion and metastasis. Therefore, targeting TAMs may improve the efficacy of CAR T cells against solid tumors. Ruella and colleagues recently devised a strategy to deplete tumor-promoting macrophages with macrophage-targeted CAR T cells. This approach was efficacious in a mouse model of Hodgkin lymphoma and led to the establishment of long-term immunological memory (115).

Myeloid derived suppressor cells (MDSCs) are another immunosuppressive cell type found in solid tumors that can dampen CAR T cell function. MDSCs express arginase and indoleamine, which metabolize amino acids that are essential for effector T cell activation and proliferation (116). Accordingly, Burga et al. demonstrated that depletion of GR1^+^ cells (targeting immunosuppressive tumor-associated neutrophils and MDSCs) augmented the ability anti-carcinoembryonic antigen CAR T cells to reduce colorectal cancer liver metastases (117). MDSCs also produce high levels of reactive oxygen species, which may impair the cytotoxic ability and proliferative capacity of CAR T cells (118). To overcome this oxidative stress, CAR T cells have been modified to express the anti-oxidant enzyme catalase into the local environment and this modification significantly improves their anti-tumor activity (119).

T_REGS_ are well-documented suppressors of T cell function capable of inhibiting anti-tumor activity through multiple mechanisms, including cell-cell contact inhibition, sequestration of IL-2 and the production of immunosuppressive cytokines such as TGF-β and IL-10 (120). Although these cells promote the growth and metastasis of tumors, they are difficult to directly deplete due to the lack of specificity of targeting agents, and the potential to induce autoimmune diseases when global disruption approaches are used (121). Given the high level of TGF-β produced by T_REGS_, MDSCs, and tumor cells, blocking TGF-β signaling through overexpression of a dominant-negative TGF-β receptor on adoptively-transferred T cells may improve their anti-tumor potency (122, 123). Overexpression of dominant-negative TGF-β receptor II on CAR T cells results in enhanced T cell proliferation, cytokine production, *in vivo* persistence and ability to eradicate tumors in mouse models of aggressive human prostate cancer (124).

Many types of cells including tumor cells, fibroblasts, endothelial cells and immune cells produce the lipid-signaling molecule prostaglandin E2 (PGE_2_) by activation of cyclooxygenase (COX)-2 and prostaglandin E synthase. PGE_2_ enhances tumor progression by stimulating multiple pathways, including those that mediate angiogenesis and immunosuppression (125). For example, PGE_2_ plays a significant role in the suppression of effector T cells and the attraction of T_REGS_ and MDSCs. PGE_2_ and adenosine activate protein kinase A (PKA), which then inhibits antigen receptor –triggered T cell activation. PGE_2_ is also known to cooperate with adenosine in the dampening of immune responses mediated by T_REGS_ (126). Recently, Newick et al. engineered CAR T cells to produce a small peptide that inhibits the association of PKA with ezrin, thus reducing the negative effects of PKA on TCR activation (127). This PKA inhibitor ameliorated the immunosuppressive actions of both adenosine and PGE_2_, resulting in increased CAR T cell trafficking, tumor cell cytotoxicity, and pro-inflammatory cytokine production (127).

### Enhancing the metabolic fitness of Car T cells

Immune cell function and metabolism are impacted by the solid tumor microenvironment. Glucose utilization is heterogeneous within the tumor and associated with perfusion, with lesser-perfused regions of the tumor displaying higher glucose metabolism (128). Both proliferating tumors and effector T cells responding to antigen challenge rely primarily on aerobic glycolysis to fuel expansion, creating competing demands for metabolites within nutrient-poor regions of the tumor (129). This competition for nutrients, metabolites and oxygen (O_2_) is thought to impact T cell metabolism, limit T cell-mediated anti-tumor efficacy and contribute to T cell exhaustion and cancer progression (130–132). Stabilization of HIF-1α drives glucose uptake, induces production of S-2-hydroxyglutarate (S-2HG) and consequential epigenetic remodeling as well as increased expression of IL-2, which potentiates CD8^+^ T cell mediated anti-tumor activity (133, 134). However, under O_2_ and glucose limiting conditions, reduction of HIF-1α expression may enhance T cell function (135). In a recent study, CD8^+^ TILs isolated from clear cell renal cell carcinoma (ccRCC) were shown to exhibit an impaired ability to consume glucose, mitochondrial fragmentation and hyperpolarization, as well as increased production of ROS (136). Because ccRCC develops a unique pathological pseudo-hypoxic response [reviewed in 137), with increased aerobic glycolysis and vascularization, it is tempting to speculate that the altered tumor microenvironment in ccRCC may have contributed to these observed defects in ccRCC CD8 TIL metabolism (136). Likewise, hypoxic areas within solid tumors are often negatively correlated with patient survival and thought to promote tumor metastasis and resistance to radiotherapy (138–140). Another metabolic checkpoint in the tumor microenvironment regulating immune modulation is amino acid limitation (129). For example, degradation of L-arginine by MDSCs in the tumor microenvironment can lead to reduced expression of CD3ζ and impaired T cell responses (141). In contrast, increased levels of arginine shift T cell metabolism to oxidative phosphorylation and increase central memory differentiation (142).

Activation, growth, proliferation, effector and memory function, and return to homeostasis are linked to the metabolic profile of the T cell (131). T cell subsets differently metabolize nutrients and regulation of nutrient availability can influence T cell differentiation as well as fate (129). Naïve T cells are metabolically quiescent and rely on glucose, fatty acids and amino acids as fuel sources for oxidative phosphorylation (143, 144). T_CM_ cells maintain spare respiratory capacity through oxidation of fatty acids in mitochondria which allows for a rapid recall of the memory response upon antigen re-challenge (145, 146). In contrast, effector T cells, like tumor cells, rely on aerobic glycolysis to provide energy, metabolic intermediates for rapid cell growth and NAD^+^/NADH to maintain redox balance (147); although under metabolically challenging conditions CD8^+^ TILs can partially preserve effector function by catabolizing fatty acids (135). Glutamine is also essential for effector function (148). After conversion to α-ketoglutarate, glutamine can serve as a TCA intermediate or contribute to the citrate pool. Similarly, altering metabolism can impact T cell phenotype; restraining glycolysis, AKT, and mTOR activity or enhancing STAT3 or Wnt/β catenin signaling can arrest T cell development and retain T_CM_ differentiation, which are associated with enhanced T cell persistence and may promote the efficacy of adoptive cell therapy (60, 149–152).

Different types of co-stimulatory endodomains incorporated into a CAR can differentially program T cell metabolism and mitochondrial biogenesis (153). This indicates that the fate of CAR T cells toward memory or effector differentiation can be directed, as cells expressing CARs with 4-1BB signaling domains have enhanced mitochondrial biogenesis and fatty acid oxidation, while CARs with CD28 signaling domains have enhanced aerobic glycolysis (i.e., Warburg metabolism) (153). Therefore, in addition to being able to direct CARs to virtually any cell surface structure on tumor cells, we also have the potential to engineer these lymphocytes to be resistant to the tumor microenvironment by specifying their metabolic program. Alternatively, host pre-conditioning strategies involving the treatment of tumors with HIF blocking agents or metabolic enzymes may represent a promising strategy to limit the metabolic flexibility of tumors as well as the localization of inhibitory immune cells (154). This would allow CAR T cells to function in a more nutrient replete and less suppressive tumor microenvironment.

### Engineering Car T cell resistance to immune checkpoint inhibitors

Tumors cells can also directly modulate effector T cell activation by expression of inhibitory signals that block T lymphocyte activation and function, thus preventing immune control of tumor growth (155). In addition to secreting immunosuppressive cytokines, tumor cells or other cells in the tumor microenvironment express a number of proteins on their surface that are capable of inactivating CAR T cells. These include PD-1 ligands, PD-L1 (B7–H1), and PD-L2 (B7-DC), all belonging to the B7 receptor superfamily. Other B7 family members, such as B7–H3 and B7–H4, and the unrelated receptors herpes virus entry mediator (HVEM), inhibitory receptor Ig-like transcript-3 and−4 (ILT3 and 4) are also abundantly expressed in the solid tumor microenvironment [reviewed in 156)]. Furthermore, by providing a persistent source of antigen while avoiding clearance, tumors potentially promote T cell exhaustion. As discussed above, checkpoint blockade has been a successful approach to sustain T cell function, and blockade of inhibitory receptors such as T-cell membrane protein-3 (TIM-3), lymphocyte-activation protein-3 (LAG-3), T cell Ig and ITIM domain (TIGIT), cytotoxic T lymphocyte-associated antigen 4 (CTLA-4), and programmed death-1 (PD-1) or their cognate ligands are being tested in clinical trials to reverse or prevent exhaustion [reviewed in (47)]. The upregulation of these receptors has been previously reported to abrogate the persistence and activity of the anti-tumor response of CAR T cells (155). Accordingly, John et al. reported that combining anti-Her2 CAR T cells and PD-1 blocking antibodies enhances tumor growth inhibition in association with decreased frequencies of GR1^+^ CD11b^+^ MDSCs (157). Strategies in which CAR T cells are engineered to secrete immune checkpoint inhibitors such as anti-PD-L1 (110), and -PD-1 (158) antibodies or PD-1-blocking single-chain variable fragments (112) possess the advantage of increasing the local delivery of these agents to the tumor microenvironment, while avoiding toxicities associated with systemic checkpoint blockade. Co-expression of a dominant-negative PD-1 receptor with mesothelin-targeted CAR T cells has also been shown to render these cells resistant to PD-1-induced inhibition and to significantly improve their *in vivo* anti-tumor efficacy following a single administration (155). The Clustered Regularly Interspaced Short Palindromic (CRISPR)/CRISPR associated protein 9 (Cas9) provides a robust and multiplexable genome editing tool that permits knock-out of inhibitory receptors (Figure 2). This system can be used to knock-out PD-1 and CTLA-4 on allogeneic universal CAR T cells (159). Finally, it is intriguing to consider the possibility of directing CAR transgenes to specific genomic loci encoding inhibitory receptors using recently developed viral and non-viral technologies (160, 161).

## Concluding remarks

Many pre-clinical studies indicate that adoptive cell transfer therapy with autologous T cells is a powerful approach for the treatment of cancer. In contrast to the recent FDA approvals of CAR T cells in hematologic malignancies, the effectiveness of this approach for a variety of more common non-hematopoietic cancers is much lower. As was underscored in this review, CAR T cells may hold great promise for the treatment of solid tumors; these malignancies have a high-unmet medical need and are generally considered incurable with present therapies. However, the achievement of complete and durable remissions for patients with non-hematopoietic cancers will require optimization of CAR T cells in the areas of improving antigen targeting, enhancing T cell trafficking, bolstering intrinsic T cell potency and arming these lymphocytes to do battle in the face of multiple immunosuppressive barriers imposed by the solid tumor microenvironment. Both current and future advances in cellular engineering, site-specific genome editing and synthetic biology will undoubtedly bolster the safety, reliability and efficacy of CAR T cell therapy for a variety of diseases. Thus, while there are currently some detours on the road to clinical success, CAR T cells are on the fast track to becoming a potentially curative modality for many different cancers.

## Author contributions

KL, RY, and JF conceptualized, wrote, and edited the manuscript. AB, MD, JM, SL, DD, and BL provided feedback and edited the manuscript.

### Conflict of interest statement

RY, MD, JM, SL, BL, and JF hold patents related to CD19-directed CAR T cell therapy. RY, MD, JM, SL and JF receive research funding from Novartis Pharmaceutical Company. RY, MD, SL and JF also receive research funding from Tmunity Therapeutics Incorporated. BL is a co-founder and equity holder in Tmunity Therapeutics, is a consultant for Novartis and CRC Oncology, and serves on the Scientific Advisory Boards of Avectas, Brammer Bio, Cure Genetics, and Incysus. The remaining authors declare that the research was conducted in the absence of any commercial or financial relationships that could be construed as a potential conflict of interest. The reviewer DD and handling Editor declared their shared affiliation at the time of review.
